# Diagnosis of Gastroesophageal Reflux Disease Using Real-time Magnetic Resonance Imaging

**DOI:** 10.1038/srep12112

**Published:** 2015-07-15

**Authors:** Shuo Zhang, Arun A. Joseph, Lisa Gross, Michael Ghadimi, Jens Frahm, Alexander W. Beham

**Affiliations:** 1 Biomedizinische NMR Forschungs GmbH am Max-Planck-Institut für biophysikalische Chemie, Göttingen, Germany; 2 Department of Surgery, University Medical Center, Göttingen, Germany

## Abstract

A small angle (His angle) between the oesophagus and the fundus of the stomach is considered to act as flap valve and anti-reflux barrier. A wide angle results in dysfunction of the oesophagogastric junction and subsequently in gastroesophageal reflux disease (GERD). Here, we used real-time magnetic resonance imaging (MRI) at 50 ms resolution (20 frames per second) in 12 volunteers and 12 patients with GERD to assess transport of pineapple juice through the oesophagogastric junction and reflux during Valsalva. We found that the intra-abdominal part of the oesophagus was bended towards the left side resulting in an angle of 75.3 ± 17.4, which was significantly larger during Valsava (*P* = 0.017). Reflux and several underlying pathologies were detected in 11 out of 12 patients. Our data visualize oesophagogastric junction physiology and disprove the flap valve hypothesis. Further, non-invasive real-time MRI has considerable potential for the diagnosis of causative pathologies leading to GERD.

Gastroesophageal reflux disease (GERD) is a common disease with a prevalence of 10–20% in Western countries and about 5% in Asia resulting in 8.9 million outpatient clinic visits in 2009^1^. Typical symptoms include chronic or episodic heartburn (pyrosis), acid regurgitation, and mucosal injury in the lower oesophagus. From a histical view, the physiology, pathopysiology and diagnosis of GERD was driven by the technical methods available at time. Initially, radiological findings such as hiatal hernia were thought to be causative for GERD. Invention of (flexible) endoscopy and oesophageal pH-monitoring introduced new indicators such as mucosal inflammation and excessive exposure of the lower oesophageal mucosa to pH < 4^2^,^3^. To date, substantial evidence has been gathered that GERD is a complex clinical entity caused or aggravated by various additional factors including gastroesophageal anti-reflux barrier dysfunction, oesophageal hypersensitivity, and poor oesophageal and/or gastric motility^4^.

Current diagnosis of GERD relies on endoscopy and pH monitoring. Endoscopy is capable of detecting advanced oesophagitis, but lacks sensitivity in determining pathological reflux, which is a particular limitation as most patients with GERD do not display macroscopic erosions. In addition, endoscopy led to the view that an anatomic flap valve is formed by the angle of His preventing reflux. The subsequent introduction of pH monitoring slightly increased the sensitivity and specificity, but could not eliminate false positives and false negatives due to the fact that 2/3 of symptomatic reflux episodes are non-acidic^5^. Moreover, the procedure requires discontinuation of acid-suppressive medication and may thus aggravate clinical symptoms. On the other hand, the general use of X-ray or CT imaging techniques is precluded because of the exposure to ionizing radiation and the limited access to soft-tissue contrast and temporal dynamics during pathophysiological swallowing conditions. Taken together, the current diagnostic tools may suffer from limited sensitivity, partial invasiveness and technical complexity.

Recent advances in magnetic resonance imaging (MRI) led to the development of an ultrafast, robust, and accurate technique for monitoring the dynamics of physiological processes in real time^6^,^7^. The use of a motion-insensitive spatial encoding strategy in combination with a high degree of data undersampling and iterative image reconstruction by regularized nonlinear inversion has made it possible to perform real-time MRI at a temporal resolution of up to 20 ms per frame. As a consequence, this non-invasive approach has already shown great promise for cardiovascular applications^8^,^9^,^10^ as well as for characterizing complex motions during swallowing, speech production and brass playing in the oropharyngeal area^11^,^12^,^13^. Moreover, a variant of the high-speed MRI technique allows for measuring fluid flow in real time and has successfully been applied to study cardiac blood flow under normal physiological conditions and during Valsalva^14^,^15^,^16^. In this article, we report our first experience of using real-time MRI for the diagnosis of GERD.

## Results

### Feasibility of Real-time MRI

Real-time MRI of gastroesophageal swallowing was feasible in all 24 subjects. For the current examination setup, no subject noticed or exhibited any problems with swallowing in a supine position.

**Figure 1:**
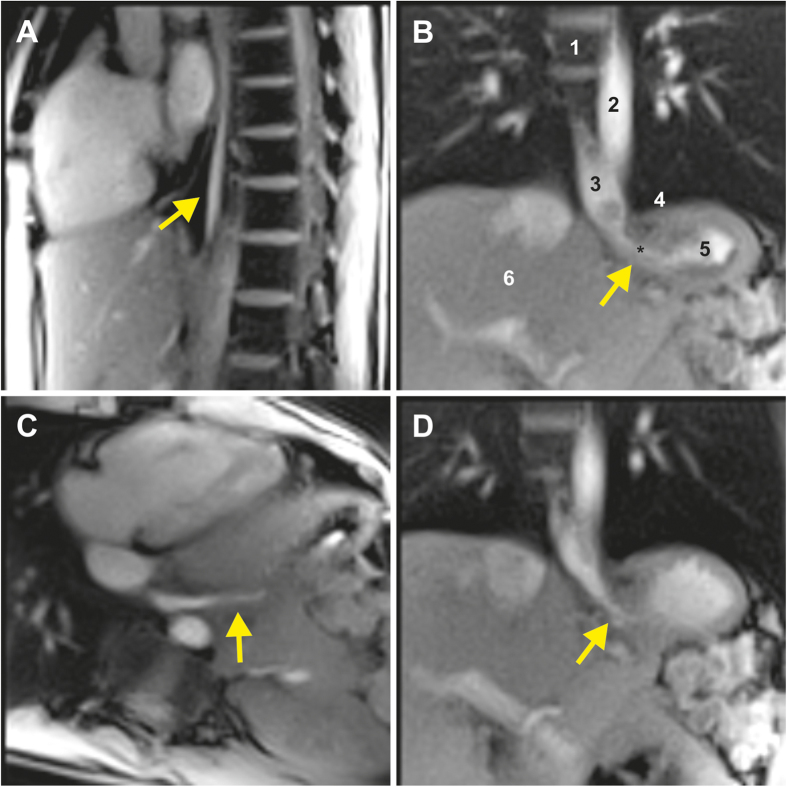
Real-time MRI of normal gastroesophageal swallowing in a 26-year-old healthy subject. Individual images (50 ms acquisition time, 2 × 2 mm^2^ resolution, 8 mm section thickness) were selected from corresponding movies (S3 to S6 in Supplementary Appendix) in (**A**) sagittal, (**B**) coronal oblique, (**C**) transversal oblique, and (**D**) coronal double-oblique planes along the directions of bolus transport (arrows). 1: spinal cord, 2: descending aorta, 3: lower oesophagus, 4: diaphragm, 5: stomach, 6: liver.

After initial localization scans, four real-time MRI measurements were performed to identify the oesophagogastric junction and to monitor the bolus transport. These included a sagittal, coronal oblique, transversal oblique and coronal double-oblique section orientation, consecutively. Each movie recording was prescribed perpendicular to the previous one and was measured with one swallowing action. The sagittal section was placed through the oesophagus, while the others were centered at the level of the diaphragm. In general, peristaltic bolus transport in the lower oesophagus, i.e. bolus accumulation at the lower oesophageal sphincter followed by its injection into the stomach, was clearly visualized by real-time MRI (Table S1 in the Supplementary Appendix). Figure 1A–D show selected images (single frames) from 4 corresponding movies (Supplementary Movies S3 to S6) of a 26-year-old female, where arrows indicate the bolus transport from the oesophagus to the stomach through the sphincter. In general, the coronal orientations provided a comprehensive view of the most important anatomical structures, from which all functional parameters were obtained.

**Figure 2:**
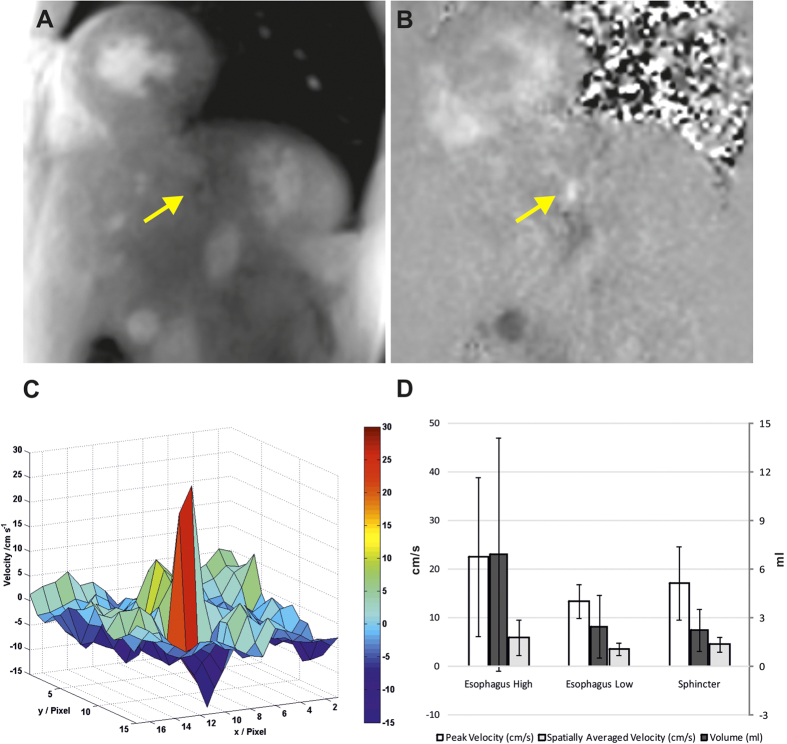
Real-time flow MRI of normal bolus transport through the lower oesophageal sphincter perpendicular to the imaging plane (same subject as in Fig. 1). (**A**) Magnitude image and (**B**) phase-contrast flow map (6 mm section thickness, velocity sensitivity 60 cm s^−1^) selected from corresponding real-time flow MRI movies (S7 and S8 in Supplementary Appendix) and (**C**) corresponding color-coded two-dimensional velocity distribution. Arrows indicate bolus transport through the lower oesophageal sphincter into the stomach. (**D**) Mean values averaged across 12 healthy subjects for peak velocities and mean velocities spatially averaged over the bolus transport lumen (in cm s^−1^) as well as for flow volumes (in ml) at three different levels along the oesophagus.

Subsequent real-time flow measurements were performed at three different levels including the sphincter, lower oesophagus before the sphincter, and higher oesophagus at approximately the level of the aortic root. For each flow measurement the imaging plane was prescribed perpendicular to the oesophagus, i.e. the flow direction. Figure 2A (Supplementary Movie S7) and 2B (Supplementary Movie S8) present a selected magnitude image and phase-contrast map during bolus pass at the lower oesophageal sphincter. In general, through-plane flow MRI measurements are accomplished by combining two images with and without a velocity-encoding magnetic field gradient perpendicular to the flow direction, i.e. usually in the same direction as the MRI slice-selection gradient. The movement of the water proton spins along this gradient leads to a differential phase information which is directly proportional to the velocity. This information is obtained in a phase-difference image referred to as phase-contrast map. Because the velocity of a fluid bolus through a particular location such as blood through the aorta or a swallow through the lower esophageal sphincter changes with time, i.e. before arrival, during passage and after completion of the bolus, high-speed variants of phase-contrast flow MRI techniques^14^,^15^,^16^ have been developed using the same principles as for morphological real-time MRI.

The relative phase in a phase-contrast map as shown in Fig. 2B is proportional to velocity, so that it may be translated into a two-dimensional velocity distribution. A corresponding representation of flow through the oesophageal sphincter is illustrated in Fig. 2C depicting color-coded velocities ranging from −15 cm s^−1^ (blue) to +30 cm s^−1^ (red). Figure 2D shows the variability of peak velocities, mean velocities spatially averaged over the bolus transport lumen, and flow volumes at three levels averaged across all healthy subjects. In most cases the intensity changes in the phase-contrast maps, which are directly proportional to changes in flow velocity, were well distinguishable. However, in about 20% of the measurements, air in the oesophagus and deformation of the oesophagus during swallowing complicated the definition of the bolus transport area and thus hindered the flow quantification. Moreover, in the higher (and lower) oesophagus the broadened spatiotemporal fluid distribution of a swallow led to rather consistent peak velocities, but hampered a meaningful calculation of mean velocities (i.e., yielding large standard deviations). This situation is improved for measurements at the oesophageal sphincter, where most of the fluid is re-assembled while still closed. However, the presence of strong blood flow in neighboring vessels such as the descending aorta eventually caused image artifacts, which distorted a clear depiction of the target area during bolus transport. For highly undersampled radial MRI acquisitions^6^ as used here, these artifacts mainly refer to “streakings” which originate from image regions with sharp borders and most pronounced intensity variations as, for example, found for arterial blood flow during systole. Similar problems may be caused by resonance offset effects due to water/fat contributions or focal distortions of the magnetic field homogeneity (e.g., susceptibility differences). For the present study, flow measurements were therefore only performed in healthy volunteers, while recent improvements of the real-time phase-contrast flow MRI technique^17^ now allow for more robust flow measurements through the oesophageal sphincter in patients with GERD.

At the end of each examination, subjects were instructed to perform a Valsalva maneuver (increase of intrathoracic pressure) for at least 10 s during real-time MRI. This was repeated for at least two previously defined imaging orientations, typically in a transversal and coronal double-oblique plane (Supplementary Movies S9 and S10).

### Healthy Subjects

Real-time MRI revealed normal swallowing dynamics and bolus transport through the oesophagogastric junction with no distortion for all 12 healthy volunteers. None of the subjects were seen with reflux during Valsalva maneuver.

Functional parameters were obtained from the morphological real-time image series during swallowing and free breathing. In addition, the His angle was also measured during the Valsalva maneuver, when it turned out to be significantly larger than during swallowing (*P* = 0.0168).

### Patients

**Figure 3:**
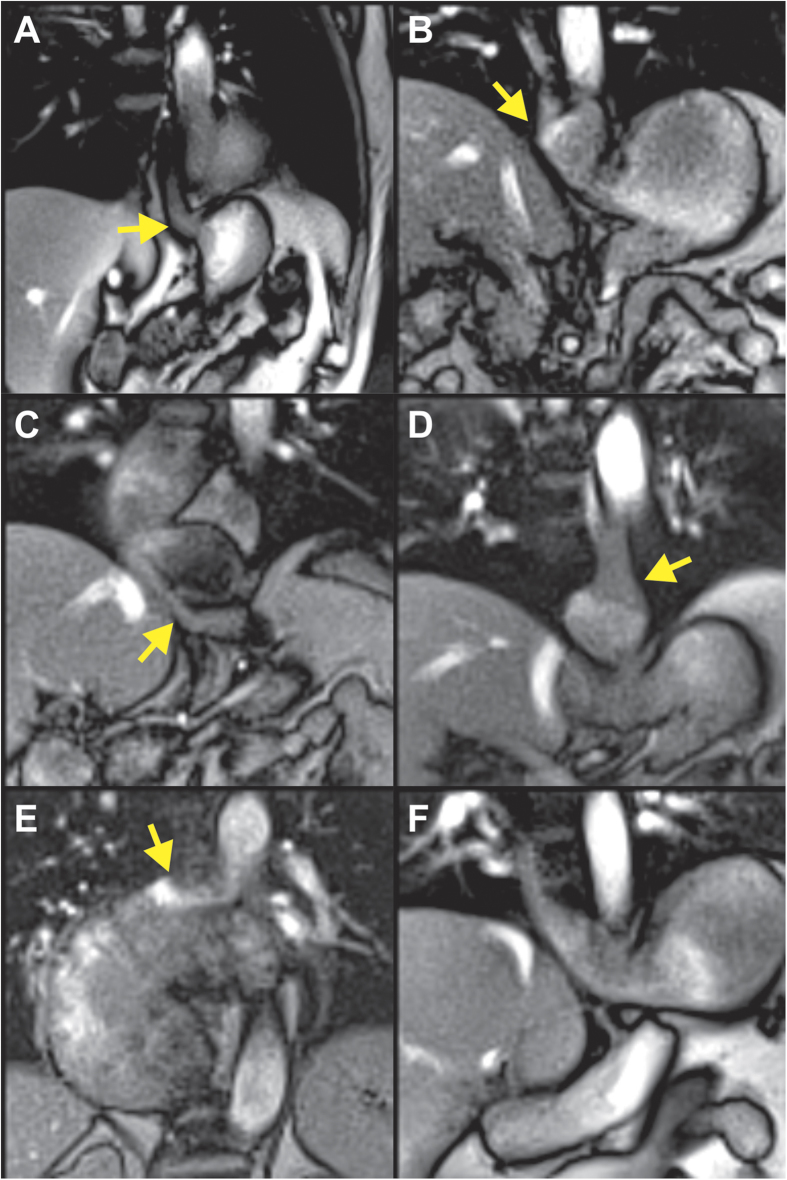
Real-time MRI of gastroesophageal swallowing in patients with (**A**) reflux, (**B**) hiatal hernia, (**C**) achalasia, (**D**) telescoping oesophagus after fundoplication, (**E**) thoracic stomach, and (**F**) functional heartburn without reflux evidence. Individual images (50 ms acquisition time, 2 × 2 mm^2^ resolution, 8 mm section thickness) were selected from corresponding movies (S11 to S17 in Supplementary Appendix). Arrows indicate (**A**,**B**) bolus regurgitation from the stomach into the oesophagus during Valsalva maneuver and (**C**–**F**) bolus transport from oesophagus into the stomach (**C**–**F**).

Twelve consecutive patients who presented themselves in our surgical outpatient clinic were included in this study. Gastroesophageal reflux could be identified in 11 out of 12 patients using real-time MRI. Regurgitation during the Valsalva maneuver could be well visualized in all 11 cases. Figure 3A presents a typical example, where the swallowed pineapple juice (high-intensity signal) returned from the stomach to the oesophagus through the sphincter. The image (single frame) was selected from the corresponding real-time MRI recording shown in Supplementary Movie S11.

In addition to gastroesophageal reflux, three gastric hernias, one achalasia, one telescoping stomach after fundoplication and one thoracic stomach were identified among the 11 affected patients. For all patients with hernias, the hiatal oesophagus led to a functional inhibition of the bolus outflow from the hernial part of the stomach, which was cranial of the diaphragm, to the lower part of the stomach below the diaphragm. An example is shown in Fig. 3B and Supplementary Movie S12, where the hiatal hernia is formed only during the Valsalva maneuver, but was not present during swallowing (Supplementary Movie S13). For the patient with achalasia, a wide oesophagus (7.0 cm) was identified with almost no visible peristaltic movement during swallowing. The transport of the bolus was limited to a very narrow passage through the sphincter (Fig. 3C and Supplementary Movie S14). In this patient clinical manometry was technically not possible. The patient with telescoping stomach had undergone fundoplication two years earlier with absence of symptoms for the initial 6 months. Real-time MRI revealed a filiform stenosis, located 2.8 cm distal to the sphincter at the side of fundoplication. It led to inhibition of the gastric clearance between the sphincter and the fundoplication (Fig. 3D and Supplementary Movie S15). Figure 3E and Supplementary Movie S16 show a patient with complete dislocation of the stomach in the thorax and a deviation of the oesophagogastric junction.

It should be noted that all 11 patients with visible oesophageal regurgitation during Valsalva maneuver had DeMeester scores >14.7, indicating gastroesophageal reflux. One patient with no visible reflux or associated pathological findings in real-time MRI had a low DeMeester score (Table S2 in Supplementary Appendix) and also no gastroscopic evidence for reflux (Fig. 3F and Supplementary Movie S17).

**Figure 4:**
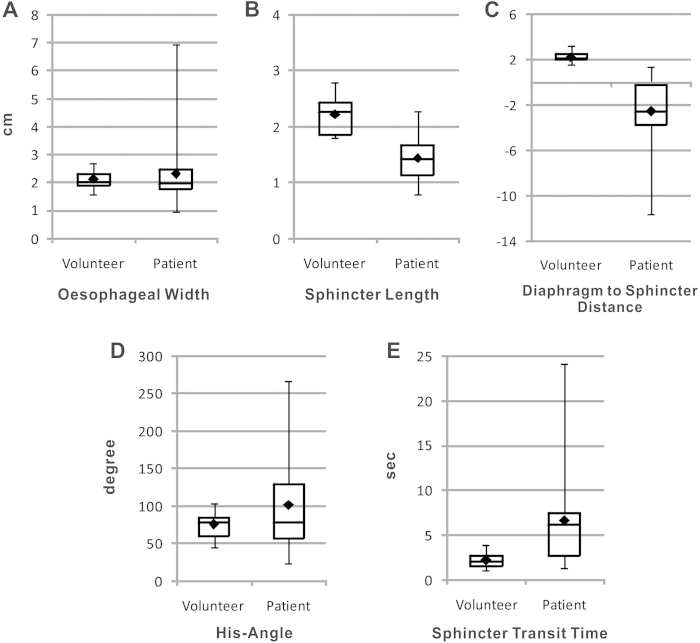
Comparison of clinical real-time MRI findings in patients (n = 12) and healthy volunteers (n = 12). Negative values of the diaphragm-to-sphincter distance indicate that the diaphragm is below the sphincter.

When comparing functional parameters between healthy volunteers and patients, diaphragm-to-sphincter distance, sphincter length, and sphincter transit time were significantly different (P < 0.05), as shown in Fig. 4. In addition, ROC analysis provided evidence that the parameters distance (ROC AUC = 0.000), sphincter length (ROC AUC = 0.089) and sphincter transit time (ROC AUC = 0.818) discriminate volunteers from patients, but not His angle (ROC AUC = 0.568) and oesophageal width (ROC AUC = 0.461) (S18 and S19).

## Discussion

In this study we established a novel method to assess the lower oesophageal junction during swallowing. Most importantly, real-time MRI offers a new perspective for the function of the anti-reflux barrier, because it disproves the current view of an anatomical flap valve formed by the angle of His. Moreover, this fast and non-invasive technique without harmful side effects allows for a robust anatomic visualization and functional assessment of gastroesophageal reflux in patients with GERD, while causative pathologies can be identified at time.

Gastroesophageal regurgitation results from a broad spectrum of underlying pathologies such as oesophageal motility disorders^18^,^19^,^20^, failure of the oesophagogastric junction and impaired gastric emptying. In particular, the oesophagogastric junction is considered to function as an anti-reflux barrier made up of three major components: lower oesophageal sphincter, crural diaphragm and anatomical flap valve. In adults, the lower oesophageal sphincter is composed by a 3–4 cm segment of conically contractive circular smooth muscle at the distal end of the oesophagus. In line with high-resolution manometry data^21^ we found that the sphincter length was significant lower in patients with GERD. The right crus of the diaphragm surrounds the distal oesophagus forming a sling and high-pressure zone of the lower oesophagus^22^,^23^, but with real-time MRI no evaluation of the crus was possible. Last, an anatomical flap valve is considered at the distal end of the oesophagus and is represented by the His angle^5^.

While current diagnostic procedures of GERD are subject to a variety of artefacts and pitfalls, real-time MRI offers excellent soft-tissue contrast with high motion robustness, negligible image artefacts and no ionizing radiation. For each subject the standardized procedure for coil setup and individual adjustment led to a total in-room time of less than 40 min, comprising 8 to 10 swallows (i.e., real-time movies) in different imaging planes. The proposed acquisition technique may be implemented on existing clinical MRI scanners without any hardware modification. However, online visualization of the images requires a parallelized version of the reconstruction algorithm and a GPU-based computer that by-passes the conventional reconstruction pipeline^10^.

Based on the present results, real-time MRI offers clinically relevant access to the dynamics of gastroesophageal swallowing. In particular, both morphological and functional information about bolus transport along the lower oesophagus as well as through the oesophagogastric junction into the gastric antrum is provided. Under real-time MRI, we observed that for healthy volunteers the descent of the stomach during a Valsalva maneuver resulted in a significantly larger His angle than during swallowing. In addition, there was no statistical difference of the His angle between healthy volunteers and patients with gastroesophageal reflux. These findings are contradictory to the hypothesis that a smaller His angle forms an anatomical anti-reflux barrier by a flap valve mechanism^5^,^20^. In fact, such observations may have been caused by interference of the endoscope that compromises the normal bending of the oesophagus and affects an accurate measurement of the His angle. On the other hand, in our cohort of patients the observation of a significantly shorter sphincter length dislocated superior to the diaphragm and a correspondingly longer bolus transit time than in healthy volunteers represent new findings that may be correlated to reflux, although more detailed studies are needed to confirm these results.

The current lack of a diagnostic gold standard presents a clinical dilemma in treating patients with reflux symptomatology. Moreover, invasive testing appears to be over-utilized, given the relatively small risk of misdiagnosis based upon an accurate patient history. So far, patients with GERD are often treated by lifestyle modifications, antacids, alginates or acid inhibitors. Proton pump inhibitor (PPI) represents the cornerstone in treatment of all manifestations and is typically followed by an initial endoscopy. However, although PPI provides symptomatic relief from heartburn and regurgitation in most cases, the potential long-term adverse effects of anti-reflux medications are still unknown^24^. For patients who do not show response to acid inhibition, additional diagnostic work-up is required. This commonly includes reflux monitoring with combined pH-impedance measurement, for which the PPI treatment has to be stopped. Surgery is indicated, if troublesome symptoms, particularly regurgitation, are persistent^25^.

This study demonstrates that (i) visualization and assessment of reflux is feasible using real-time MRI, and that (ii) the accessible anatomical and physiological information is clinically valuable for the diagnosis of pathologies and subsequent surgical planning. Three patients who showed gastric hernia and functional gastric outlet defect with no response to PPI underwent fundoplication. For one patient who had servere reflux, a Nissen (360°) fundoplication^26^ was performed; for the other two patients with relatively moderate reflux a Toupet (270°) fundoplication was done^26^,^27^. The patient with achalasie received endoscopic botox injection into the lower oesophageal sphincter. Subsequent real-time MRI revealed a reduced oesophageal diameter (5.3 cm compared to 7.0 cm before treatment), improved bolus transport from the oesophagus into the stomach, and gastric regurgitation during Valsalva maneuver. In one patient with no evidence of reflux under real-time MRI, endoscopy and reflux monitoring findings were also negative, which led to the diagnosis of oesophageal hypersensitivity^28^. Of course, to which extent real-time MRI may contribute to an early identification of these pathologies has to be further investigated^29^.

Although real-time velocity-encoded flow MRI was possible in almost all healthy volunteers, the difficulty in properly defining the transactional oesophageal lumen hindered a reliable flow quantification in patients who present with a wide range of anatomical variations. In these cases the reflux volume was also technically difficult to measure, because of the very low flow velocities during regurgitation. Furthermore, the general MRI quality strongly depends on the distance between the receive coils and the tissue of interest (i.e., the sphincter), which for obese patients leads to a reduced signal-to-noise ratio. Another limitation of this work is the small number of patients. The variety of causative pathologies in GERD requires further clinical studies to define specificity and sensitivity of real-time MRI for individual gastroesaphageal pathology.

In conclusion, our findings suggest that real-time MRI with high spatiotemporal resolution shows great promise as a safe, rapid, robust, and clinically relevant imaging technique for the diagnosis of gastroesophageal reflux disease and may represent the next technical quantum leap helping to understand GERD. Depending on future systematic studies of more patients, it is foreseeable that real-time MRI will facilitate the identification of pathologies and contribute to decision making for therapeutic treatment of patients with GERD.

## Materials and Methods

### Subjects

After developing the method for oesophagus real-time MRI assessment, we conducted a phase 1 trails to compare healthy individuals and patients with GERD. The subjects in this study included 12 healthy volunteers and 12 patients, recruited from the Departments of Surgery of the University Medical Center Göttingen. For volunteers (6 men, 6 women; age 28 ± 4 years [mean ± standard deviation]; range 24–37 years) no history or presence of gastroesophageal reflux-associated symptoms or other chronic illnesses were assured by personal medical history. Twelve consecutive outpatients with GERD were also studied. These included 6 men and 6 women with a mean age of 53 ± 15 years ranging from 20 to 73 years. The ethical board approved the study and all participants gave written informed consent before each examination.

### Real-time MRI

Subjects were examined in a supine position with use of a commercial 3 T MRI system (Tim Trio, Siemens Healthcare, Erlangen, Germany) and a thorax coil comprising 32 independent receive elements. Real-time MRI was accomplished by our previously described undersampled radial fast low-angle shot (FLASH) acquisition and regularized nonlinear inverse (NLINV) reconstruction technique^6^. The former encodes gradient-echo MRI signals along radial spokes that are equally distributed to homogeneously sample the data space. The NLINV reconstruction allows for a simultaneous calculation of the image content and its coil sensitivities using all acquired data for each frame in an iterative optimization process. The algorithm was highly parallelized using a specially designed library for efficient multi-GPU computing^2^ and implemented on a computer equipped with two processors (CPUs, SandyBridge E5-2650, Intel, Santa Clara, CA) and eight graphical processing units (GPUs, GeForce GTX TITAN, Nvidia, Santa Clara, CA). This customized computer was fully integrated into the architecture of the commercial MRI system, where it is invisible to the user and does not need any user interference. It effectively bypasses the standard reconstruction pipeline, while storing reconstructions as conventional DICOM images in the regular databank^10^. Current online reconstruction and display rates are about 20 frames per second for real-time images and flow-encoded magnitude images and phase-contrast maps.

During dynamic imaging, pineapple juice served as an oral contrast agent because of its T1 shortening effect due to an inherent concentration of paramagnetic manganese ions^11^. After the onset of each real-time measurement a bolus of 10 ml pineapple juice was manually injected into the subject’s mouth by one operator (A.J.) standing next to the MRI magnet. The fluid was delivered with the use of a 50 ml syringe connected to a conventional flexible infusion tube with a diameter of 3 mm, the end of which was fixed to the subject’s mouth. The end of the bolus administration was cued by the operator, after which the volunteer performed a self-controlled voluntary swallow in a natural manner at a comfortable rate. The bolus was given once for each real-time MRI recording, which lasted for about 25 s.

### Study Protocol

For real-time MRI, mildly T1-weighted images were continuously acquired with the following parameters: RF-spoiled radial FLASH, repetition time TR = 2.00 ms, echo time TE = 1.28 ms, flip angle 5°, field of view 192 × 192 mm^2^, in-plane resolution 2.0 × 2.0 mm^2^, and slice thickness 8 mm. Individual images were obtained from 25 radial spokes resulting in an image acquisition time of 50 ms and a true temporal resolution of 20 frames per second (fps) with no data sharing or interpolation.

Real-time MRI of through-plane flow was based on the adaptation of velocity-encoding phase-contrast principles. For this purpose, the radial FLASH acquisition was combined with a motion-compensated slice-selective gradient and a bipolar flow-encoding gradient applied in every other image^14^,^15^ The imaging parameters were as follows: TR = 3.57 ms, TE = 2.56 ms, flip angle 10°, field of view 256 × 256 mm^2^, in-plane resolution 2.0 × 2.0 mm^2^, slice thickness 6 mm, and velocity sensitivity = 60 cm s^−1^. Individual images were obtained from only 7 radial spokes, so that the total acquisition time for a pair of images with and without velocity-encoding gradient was 49.98 ms. Dynamic phase-contrast maps were therefore obtained with the same temporal resolution of 20 fps as used for morphological real-time MRI. The methods were carried out in accordance with approved guidelines.

### Image Evaluation

Functional parameters including oesophageal width, sphincter length, diaphragm-to-sphincter distance, His angle, and sphincter transit time were measured directly from the dynamic image series. In more detail, oesophageal width was measured at a position about 3 cm above the lower oesophageal sphincter, diaphragm-to-sphincter distance was measured from the lower sphincter boundary, His angle was observed where the oesophagus regularly bended toward the stomach, and sphincter transit time was defined as the time interval between bolus head and tail passing through the sphincter. Flow-related functional parameters were derived from serial phase-contrast maps and corresponding magnitude images and included peak velocities, mean velocities spatially averaged over the bolus transport lumen, and flow volumes.

The evaluations were jointly performed by one experienced surgeon and two physicists skilled in the interpretation of MR images. Analyses were based on the manufacturer’s software (Syngo B17, Siemens Healthcare, Erlangen, Germany). Before evaluation of the patient MRI data, the examiner had no information concerning the diagnosis of the patients.

### Statistical Analysis

The statistical significance of differences between healthy volunteers and patients was determined with the use of an independent *t*-test. A two-tailed P value of less than 0.05 was considered statistically significant. The analysis was carried out using SPSS software.

## Additional Information

**How to cite this article**: Zhang, S. *et al.* Diagnosis of Gastroesophageal Reflux Disease Using Real-time Magnetic Resonance Imaging. *Sci. Rep.* **5**, 12112; doi: 10.1038/srep12112 (2015).

## Supplementary Material

Supplementary Movie S3

Supplementary Movie S4

Supplementary Movie S5

Supplementary Movie S6

Supplementary Movie S7

Supplementary Movie S8

Supplementary Movie S9

Supplementary Movie S10

Supplementary Movie S11

Supplementary Movie S12

Supplementary Movie S13

Supplementary Movie S14

Supplementary Movie S15

Supplementary Movie S16

Supplementary Movie S17

Supplementary Information
