# Supplementary material for: Diagnosis of Gastroesophageal Reflux Disease Using Real-time Magnetic Resonance Imaging
Source: Sci Rep. 2015 Jul 15;5:12112. doi: 10.1038/srep12112 (PMC4648433; doi:10.1038/srep12112)
Supplement: Supplementary Information [file srep12112-s16.pdf]

Diagnosis of Gastroesophageal Reflux Disease Using Real-time Magnetic Resonance Imaging

Shuo Zhang, Arun A Joseph, Lisa Gross, Michael Ghadimi, Jens Frahm, Alexander W Beham

SUPPLEMENTARY APPENDIX

Table S1. Real-time MRI visibility of gastroesophageal swallowing.

Image orientation		Sagittal	Coronal oblique	Transversal oblique	Coronal double-oblique
Oesophageal peristalsis		√	√	(√)	√
Bolus transport	Oesophagus	Middle	Lower		Lower
	Lower oesophageal sphincter		√	√	√
	Stomach		√	√	√
Gastro-oesophageal reflux		(√)	√	√	√

√ Visible in all cases; (√) visible in limited cases.

Table S2. DeMeester scores and endoscopic findings in patients (n = 12).

Patient	Age	DeMeester score	Endoscopy
1	48	62.0	
2	58	19.6	Barrett oesophagus
3	26	14.7	
4	64	28.8	
5	68	73.9	Reflux oesophagitis I
6	20	4.3	
7	58	14.7	
8	50	150.9	Fundoplication
9	54	16.5	
10	73	26.9	
11	62	12.1	Reversed stomach
12	47	21.6	

Movie S3: Real-time MRI of normal gastroesophageal swallowing – sagittal plane. The image series at 50 ms resolution covers bolus transport of a single swallow through the lower oesophagus (arrow) of a 26-year-old healthy female (2 × 2 mm^2 resolution, 8 mm thickness). For details see text and Figure 1A.

Movie S4: Real-time MRI of normal gastroesophageal swallowing – coronal oblique plane. The image series at 50 ms resolution covers bolus transport of a single swallow through the oesophagogastric junction (arrow) of a 26-year-old healthy female (2 × 2 mm^2 resolution, 8 mm thickness). For details see text and Figure 1B.

Movie S5: Real-time MRI of normal gastroesophageal swallowing – transversal oblique plane. The image series at 50 ms resolution covers bolus transport of a single swallow through the oesophagogastric junction (arrow) of a 26-year-old healthy female (2 × 2 mm^2 resolution, 8 mm thickness). For details see text and Figure 1C.

Movie S6: Real-time MRI of normal gastroesophageal swallowing – coronal double-oblique plane. The image series at 50 ms resolution covers bolus transport of a single swallow through the oesophagogastric junction (arrow) of a 26-year-old healthy female (2 × 2 mm^2 resolution, 8 mm thickness). For details see text and Figure 1D.

Movie S7: Real-time flow MRI (magnitude images) at the lower oesophageal sphincter. The image series at 50 ms resolution covers bolus transport of a single swallow through the oesophagogastric junction (arrow) of a 26-year-old healthy

female (2 × 2 mm^2 resolution, 6 mm thickness, velocity sensitivity 60 cm s^{-1}). For details see text and Figure 1E.

Movie S8: Real-time flow MRI (phase-contrast maps) at the lower oesophageal sphincter. The image series at 50 ms resolution covers bolus transport of a single swallow through the oesophagogastric junction (arrow) of a 26-year-old healthy female (2 × 2 mm^2 resolution, 6 mm thickness, velocity sensitivity 60 cm s^{-1}). For details see text and Figure 1F.

Movie S9: Real-time MRI of the oesophagogastric junction during Valsalva maneuver – coronal oblique plane. The image series at 50 ms resolution was obtained from a 26-year-old healthy female (2 × 2 mm^2 resolution, 8 mm thickness, velocity sensitivity 60 cm s^{-1}). For details see text.

Movie S10: Real-time MRI of the oesophagogastric junction during Valsalva maneuver – transversal oblique plane. The image series at 50 ms resolution was obtained from a 26-year-old healthy female (2 × 2 mm^2 resolution, 8 mm thickness, velocity sensitivity 60 cm s^{-1}). For details see text.

Movie S11: Real-time MRI of gastroesophageal reflux in a 61-year-old male patient. The image series (coronal oblique plane) at 50 ms resolution was obtained during Valsalva maneuver (2 × 2 mm^2 resolution, 8 mm thickness). The arrow indicates the bolus regurgitation. For details see text and Figure 2A.

Movie S12: Real-time MRI of gastroesophageal swallowing in a 48-year-old male patient with gastric hernia. The image series (coronal oblique plane) at 50 ms

resolution was obtained during swallowing (2 × 2 mm^2 resolution, 8 mm thickness). Notice the absence of the hiatal hernia. For details see text and Figure 2B.

Movie S13: Real-time MRI of gastroesophageal reflux in a 48-year-old male patient with gastric hernia. The image series (coronal oblique plane) at 50 ms resolution was obtained during Valsalva maneuver (2 × 2 mm^2 resolution, 8 mm thickness). The arrow indicates the formation of the hernia during bolus regurgitation. For details see text and Figure 2B.

Movie S14: Real-time MRI of gastroesophageal swallowing in a 64-year-old female patient of achalasia. The image series (coronal oblique plane) at 50 ms resolution was obtained during swallowing (2 × 2 mm^2 resolution, 8 mm thickness). The arrow indicates the narrow pathway of bolus transport. For details see text and Figure 2C.

Movie S15: Real-time MRI of the gastroesophageal swallowing in a 68-year-old female patient with telescoping oesophagus after fundoplication. The image series (sagittal plane) at 50 ms resolution was obtained during swallowing (2 × 2 mm^2 resolution, 8 mm thickness). The upper and lower arrows indicate the sphincter and the narrow pathway of bolus transport, respectively. For details see text and Figure 2D.

Movie S16: Real-time MRI of the gastroesophageal swallowing in a 62-year-old female patient of thoracic stomach. The image series (coronal oblique plane) at 50 ms resolution was obtained during swallowing (2 × 2 mm^2 resolution, 8 mm

thickness). The arrow indicates the bolus transport. For details see text and Figure 2E.

Movie S17: Real-time MRI of the gastroesophageal swallowing in a 20-year-old female patient of functional heartburn. The image series (coronal oblique plane) at 50 ms resolution was obtained during swallowing (2 × 2 mm^2 resolution, 8 mm thickness). For details see text and Figure 2F.

Figure S18

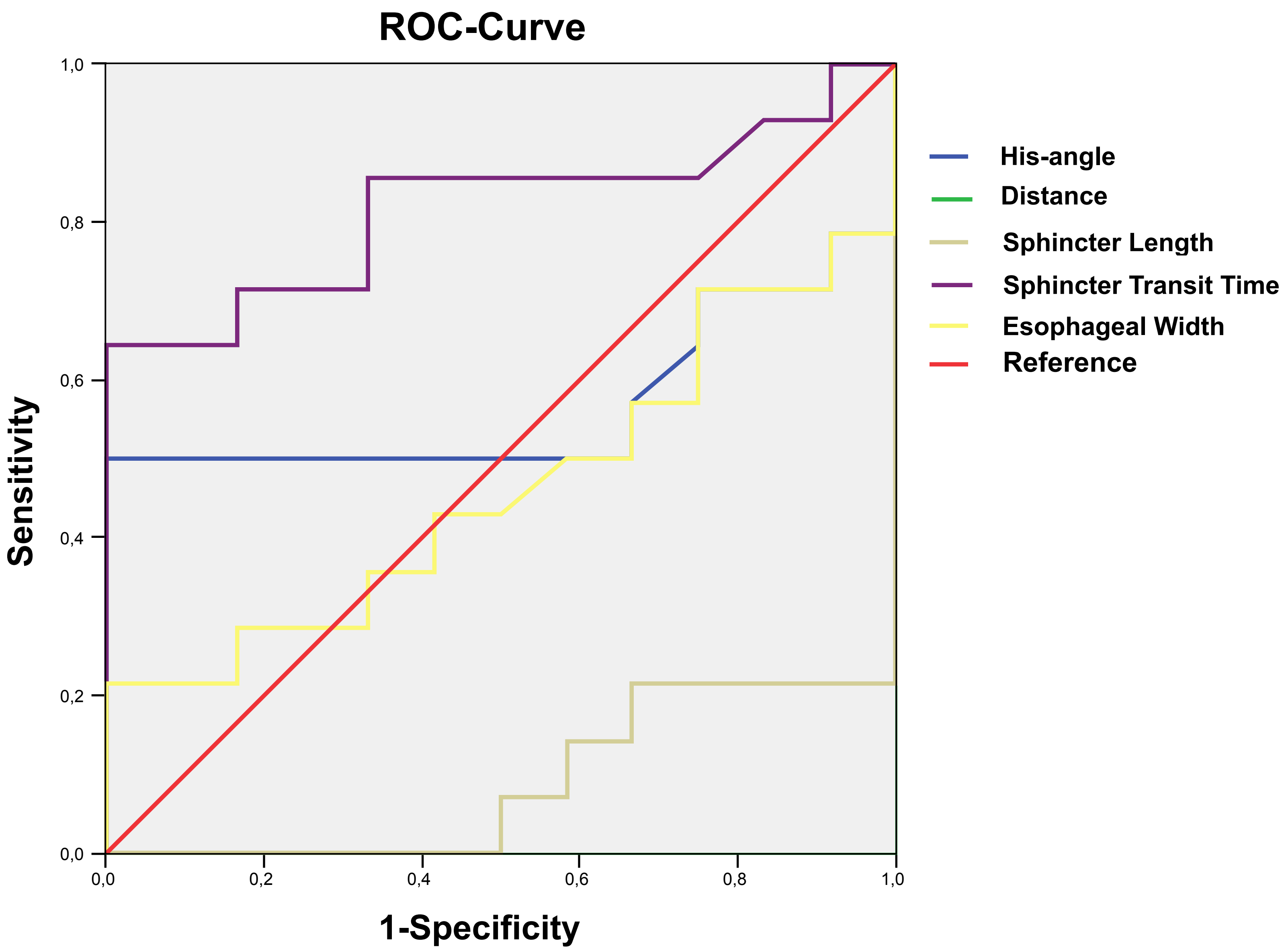

ROC-Curve
Sensitivity
1-Specificity
0,0
0,2
0,4
0,6
0,8
1,0
His-angle
Distance
Sphincter Length
Sphincter Transit Time
Esophageal Width
Reference

Table S19

Area under the curve for receiver operating characteristic (ROC)

His Angle	0.568
Distance	0.000
Sphincter Length	0.089
Sphincter Transit Time	0.818
Esophageal Width	0.461
